# Addition of Clonidine in Caudal Anesthesia in Children Increases Duration of Post-Operative Analgesia

**DOI:** 10.5812/kowsar.22517464.3393

**Published:** 2012-01-15

**Authors:** Marzieh Lak, Hasan Araghizadeh, Shahnas Shayeghi, Behroz Khatibi

**Affiliations:** 1 Trauma Research Center, Baqiyatallah University of Medical Sciences, Tehran, IR Iran; 2 Department of Anesthesiology, Baqiyatallah University of Medical Sciences, Tehran, IR Iran

**Keywords:** Analgesia, Bupivacaine, Clonidine, Anesthesia, Caudal

## Abstract

**Background:**

Pain in infancy is a developmental process. Due to the underdeveloped pain pathways in the spinal cord, the threshold of stimulation and sensation of pain is low at birth and has potential impacts on increasing the central effects of pain. Primary trauma during infancy can cause long term changes in structure and function of pain pathways that continue until adulthood. Lack of pain management in children can result in morbidity and mortality.

**Objectives:**

In this study we examined the duration of post-operative analgesia in children when clonidine is added to bupivacaine in caudal anesthesia.

**Materials and Methods:**

In this clinical trial, 40 children aged 1-8 years who were candidates for elective inguinal hernia repair were studied. Induction and maintenance of anesthesia were achieved using sodium thiopenthal, halothane and nitrous oxide. Children were randomly divided into 2 groups in a double-blind fashion, and were given caudal anesthesia with 0.125% bupivacaine (1ml/kg) alone or b bupivacaine plus 2 μg/kg clonidine. Blood pressure and heart rate were recorded peri-operatively. Analgesia was evaluated using objective pain scale (OPS) and sedation was assessed using Ramsay sedation scale (RSS). Acetaminophen was administered rectally for cases with OPS score greater than five.

**Results:**

Duration of analgesia was found to be significantly longer in the group given bupivacaine plus clonidine (mean 417.50 min vs. 162.00 min).

Peri-operative hypotension or bradycardia, post-operative respiratory depression, nausea or vomiting were not recorded in any patient.

**Conclusions:**

We concluded that addition of clonidine to bupivacaine prolongs the duration of post-operative analgesia without any respiratory or hemodynamic side-effects.

## 1. Background

Pain in infancy is a developmental process. Due to the underdeveloped pain pathways in the spinal cord, the threshold of stimulation and sensation of pain is low at birth and has potential impacts on increasing the central effects of pain. Primary trauma during infancy can cause long term changes in structure and function of pain pathways that continue until adulthood (1). Lack of pain management in children can result in morbidity and mortality (2). Due to the better comprehension of neurobiology and pharmacology of pain in the past 2 - 3 decades, use of analgesics and regional pain management techniques in children have improved significantly. Pain management protocols that use one type of pain reliever aiming to control one of the pathways of pain perception are usually less effective compared to those that use a combination of analgesics with the aim of controlling several pain pathways.

Opioids remain the mainstay for treatment of acute moderate to severe pain. But we can decrease their use by addition of other analgesics and use of techniques that target other parts of pain pathways and obtain an equal or even greater analgesia. Thus, decreasing adverse effects of opioids like nausea, vomiting, constipation, itching, respiratory depression and urinary retention (3).It should be noted that use of opioids in infants younger than 4 - 6 months can cause apnea and hypoventilation (2). Caudal anesthesia is the oldest and at present the most common epidural technique in children (4).Single dose injection in caudal anesthesia is the most effective and most prevalent form of regional block in children. This method is easy, reliable and safe especially in children weighing less than 10 kg. Single dose injection of anesthetics is suitable especially for below the navel operations (5). Caudal block along with general anesthesia is used for post-operative analgesia in infra-umbilical operations. This way, lower concentration of inhaled anesthetics is used and regaining consciousness after the operation is expedited. Caudal block is a simple method with lowest rate of complications. Bupivacaine is the most common anesthetic used for caudal block because it is relatively safe and long acting. Opioids, clonidine and ketamine have been used for caudal block with various degrees of success (6).

In a survey on members of the British Pediatric Anesthesia Society, 58% stated that they have used additives in caudal block in children in order to prolong the duration of analgesia with no increase in the rate of complications such as loss of motion. The most common additives were ketamine, clonidine, fentanyl and diamorphone (7). Clonidine is the agonist of alpha 2 adrenergic receptors. When added to local anesthetics, whether in peripheral or neuraxial block, it has many benefits without hemodynamic effects (4). However, some studies have reported that adding clonidine to bupivacaine in caudal block has had no clinical advantage over bupivacaine alone. For example, in a study conducted by Wheeler and colleagues on 30 patients aged 2-8 yrs. who were candidates for below the navel operations, no significant difference was detected between those receiving bupivacaine and clonidine and those receiving bupivacaine alone (8). Various studies have used clonidine as an additive in caudal anesthesia reporting controversial results. Clonidine in its usual dosage (1-2 µg/kg) has no considerable side effects (9). This drug has not been imported to our country yet and therefore, no study has been conducted on it in IR Iran. In this study we evaluated the effects of adding clonidine to caudal block in children undergoing elective herniorrhaphy.

## 2. Objectives

In this study we examined the duration of post-operative analgesia in children when clonidine is added to bupivacaine in caudal anesthesia.

## 3. Materials and Methods

In this randomized double-blind clinical trial, 40 ASA class I children aged 1-8 years weighing less than 20kg who were candidates for elective herniorrhaphy were selected. Patients were visited the night before the surgery. Parents were thoroughly informed about the study and gave an informed consent. Patients with a history of allergy to local anesthetics, those with coagulation disorders, spinal cord deformity, skin infection at the site of injection, and those with a history of metabolic, neurologic or cardiac diseases were excluded from the study. All patients received atropine and oral midazolam as premedication before transfer to the operating room. Patients in whom caudal block was diagnosed to be contraindicated for any reason by the anesthesiologist, were excluded from the study. Standard monitoring was performed for all patients. Anesthesia was induced using sodium thiopenthal 5-7 mg/kg and atracurium 0.5 mg/kg. Adequate dosage of halothane and a combination of nitrous oxide and oxygen with an equal proportion were used for maintenance of anesthesia.

After induction of general anesthesia, the child was placed on his/her left lateral decubitus position. Caudal block was induced using 23-guage needle and after making sure that blood or CSF was not found in aspiration, anesthetic drug was injected. In order to select the medication for caudal block randomly and in a double blind fashion, the drug was prepared by another anesthesiologist by combining bupivacaine and normal saline and only the code of the drug was given to the researcher. In group 1 bupivacaine 0.125% 1 ml/kg along with clonidine 2 µg/kg (group BC) and in group 2 bupivacaine 0.125% 1ml/kg along with normal saline (Group B) were administered. The amount of normal saline in group 2 was similar to the amount of clonidine in group 1. If caudal block was unsuccessful or if the patient required more than 0.5 MAC of inhaled anesthetic for maintaining the adequate depth of anesthesia15 min after performing the block, the patient was excluded from the study.

Patients received Ringer’s solution based on their weight and duration of operation peri-operatively and blood pressure (BP) and heart rate (HR) were recorded every 5 minutes. Any decrease in BP and HR for more than 20% compared to the pre-operative values was considered as hypotension and bradycardia and treated with quick infusion of fluids and atropine, respectively. Any increase in BP and HR for more than 20% of the pre-operative values was considered as inadequate analgesia and was treated with fentanyl 1µg/kg if necessary. After completion of surgery, patients were transferred to the recovery room awake and were evaluated in terms of pain and sedation using Objective Pain Scale and Ramsay Sedation Scale, respectively. Assessments were made immediately after the transfer and then every 15 minutes until they were ready to leave the recovery room. For pain scale greater than 5, analgesics were administered and the time of administration was recorded. After transferring to the ward, if the patient did not need analgesics in the recovery room, pain was assessed in the ward, and for pain scale over 5, analgesics were given and recorded. If the patient did not require analgesics for 24 hours, it was registered under the category of no necessity for analgesics.

The time interval between the conduction of caudal block and time of receiving the first dose of analgesic was considered as duration of post-operative analgesia. Researchers followed the ethical principles of Declaration of Helsinki in all steps and patients’ name and personal information remained confidential. Data collected were analyzed using SPSS version 11.5 software (Microsoft). Prevalence of variables was determined using statistical tests. For comparison of quantitative variables between the 2 groups, student t test and for qualitative variables Chi square test was used.

## 4. Results

In this study a total of 40 children were evaluated in 2 groups of 20 each. Two patients were excluded from the study and replaced due to having spinal cord deformity. Table 1 demonstrates the demographic characteristics of patients. The mean age of patients was 35.70 months (range 12-72) in group B and 42.30 (12-92) months in group BC and not statistically significant (P > 0.05, Table 1). The mean weight of patients was 12.1 kg in group B and 16.7 kg in group BC and not statistically significant (P > 0.05, Table 1). The mean duration of operation was 32.75 (15-50) min in group B and 40.35 (15-60) min in group BC and not statistically significant (P > 0.05, Table 2). The mean duration of anesthesia was 45.85 (30-60) minutes in group B and 55.25 (30-75) min in group BC and not statistically significant (P > 0.05, Table 2). The mean recovery time was 40.75 (25-70) min in group B and 38.55 (25-50) min in group BC and not statistically significant (P = 0.563, Table 2).

**Table 1.** The Comparison of Demographic Characteristics of Patients in the 2 Groups

	Group B ^a^	Group BC ^a^
Mean age (Month, Min-Max)	35.70 (12-72)	42.30 (12-92)
Mean weight (Kg)	12.1	16.7
Gender (Male/Female)	8.12	6.14

^a^ Abbreviations: B: Receiving bupivacaine plus placebo; BC: Receiving bupivacaine plus clonidine

**Table 2.** Comparison of Mean Duration of Anesthesia, Mean Duration of Surgery, Mean Duration of Analgesia, Sedation Score, Analgesia Score, Recovery Time and Extubation Time in 2 Groups of B and BC.

	Group B	Group BC
Mean duration of anesthesia (min)	45.85	55.25
Mean duration of surgery (min)	32.75	40.35
Mean duration of post-op analgesia (min)	162.00	417.50
Mean score of sedation (min)	2.52	3.09
Mean score of analgesia (min)	3.59	2.92
Mean recovery time (min)	40.75	38.55
Mean extubation time (min)	7.50	4.45

The mean time of extubation was 7.50 (2-15) min in group B and 4.45 (2-10) min in group BC. This difference was statistically significant (P = 0.002, Table 2). The mean score of sedation from the time of entering the recovery room till the time of receiving analgesic was 2.52 in group B and 3.09 in group BC. This difference was statistically significant (P = 0.002, Table 2). All patients in both groups required analgesics during the first 24 hours post operation. The mean score of analgesia from the time of entering the recovery room till receiving analgesics was 3.59 in group B and 2.92 in group BC. This difference was statistically significant (P = 0.002, Table 2).The mean duration of analgesia (from the time of caudal block till receiving the first analgesic drug) was 162.00 minutes in group B and 417.50 min in group BC. This difference was statistically significant (P = 0.000, Table 2).

The mean arterial pressure (MAP) before induction of anesthesia was 88.35 (75-112) mmHg in group B and 85.55 (70-98)mmHg in group BC (Table 3). The mean arterial pressure after induction of anesthesia and before induction of caudal block was 77.25 (65-100) mmHg in group B and 74.75 (62-104) mmHg in group BC (Table 3). MAP after caudal block and till the end of operation was 78.00 (67-100) mmHg in group B and 74.60 (61-85) mmHg in group BC (Table 3). The mean heart rate before induction of anesthesia was 141.95 (110-170) per minute in group B and 137.65 (107-174) per minute in group BC (Table 3). The mean HR after anesthesia induction and before caudal block was 130.45 (105-155) pulse/minute in group B and 128.30 (95-170) pulse/minute in group BC (Table 3). The mean HR after caudal block till the end of operation was 113.65 (83-135) in group B and 118.15 (93-142) pulse/minute in group BC (Table 3). None of the understudy children had any underlying diseases and nausea, vomiting, respiratory depression, blood pressure drop, bradycardia or urinary retention were not reported in any patient. None of the under study subjects required any type of therapeutic intervention.

**Table 3.** Comparison of Mean Arterial Pressure and Heart Rate before the Induction of Anesthesia, After the Induction of Anesthesia and Peri-Operatively in the 2 Groups of B and BC.

	Group B	Group BC
^a^ MAP before anesthesia induction (mmHg)	88.35	85.55
MAP after anesthesia induction (mmHg)	77.25	74.75
MAP peri-operatively (mmHg)	78	74.60
^a^ HR before anesthesia induction	141.95	137.65
HR after anesthesia induction	130.45	128.30
HR peri-operatively	113.65	118.15

^a^ Abbreviations: MAP: Mean arterial pressure, HR: Heart rate

## 5. Discussion

In this study, 40 ASA class I children aged 1-8 yrs. and weighing less than 20 kg who were candidates for elective herniorrhaphy were evaluated. We found that addition of clonidine to bupivacaine in caudal block prolongs post-operative analgesia with no side effects (417.5 min in group BC versus 162 min in group B). This finding was in accord with most of the similar previous studies. In the present study we used bupivacaine 0.125% for caudal block which had been reported to cause adequate analgesia without loss of motion (10). Caudal block with bupivacaine alone can cause adequate analgesia post-operatively but by reversing the block patients mostly need administration of systemic analgesics (11). Therefore, in this study we added clonidine 2 µg/kg to bupivacaine since in previous studies clonidine with different dosage of 1, 1.5 and 2 µg/kg had been used. When analyzing the results, it was revealed that caudal clonidine with the dosage of 2 µg/kg resulted in longer analgesia when compared with dosage of 1 and 1.5 µg/kg. Additionally, it caused no hypotension or bradycardia peri-operatively and no respiratory depression or loss of motion post-operatively (10).

Addition of clonidine in caudal block has proved to be safe in various studies. In a report published in 2008 in 3 pediatric patients who received one hundred times the standard dose of caudal clonidine, despite severe drowsiness for 24 hours, respiratory depression, decrease in oxygen saturation, need for oxygen administration or hemodynamic instability were not observed (9). This indicates the wide safety margin of caudal clonidine in children. Akin et al. in 2010 evaluated 60 ASA class I and II children aged 2-8 yrs who were candidates for herniorrhaphy and orchidopexy in 3 groups of 20 each. For post-op analgesia, all were supposed to receive caudal block with levobupivacaine. In group 1, levobupivacaine 0.25% alone, in group 2, levobupivacaine along with caudal clonidine 2 µg/kg and in group 3, caudal levobupivacaine along with intravenous clonidine 2 µg/kg were administered. When comparing the effects of caudal versus intravenous clonidine, it was found that caudal clonidine prolonged the analgesia caused by caudal levobupivacaine without causing significant side effects (12).

Our study was different from that of Akin et al. in that they used caudal clonidine in one group and intravenous clonidine with the same dose of 2 µg/kg in another group. However, our study results were in accord with theirs and in both studies those receiving caudal clonidine had the longest duration of post-op analgesia. El-Henawy et al. in 2009 performed a study on 60 children aged 6 months to 6 years and evaluated the effect of addition of clonidine or dexmedetomidine to caudal bupivacaine . They demonstrated that addition of clonidine or dexmedetomidine to caudal bupivacaine significantly prolongs the analgesia in pediatric patients undergoing infra-abdominal surgeries with no increase in the incidence of side effects. Also, they showed that clonidine and dexmedetomidine had no advantage over each other (11). Our study results were in accord with those of El-Henawy et al. However, the 2 studies were different in that El-Henawy and colleagues evaluated the effects of dexmedetomidine as an addition to the caudal drug in a separate group as well. Vetter and colleagues in 2007 conducted a study on 60 pediatric patients aged 6 months to 6 yrs. undergoing ureteral reimplantation. Patients were divided into 3 groups of 20 each. All patients underwent caudal block with ropivacaine 2% 1ml/kg. Group 1 received addition of clonidine 2 µg/kg, group 2 hidromorphone 10 µg/kg and group 3 morphine 50 µg/kg. They showed that although morphine may cause a stable primary analgesia, caudal clonidine seems to be able to cause a comparable analgesia with minimum side effects. According to this study, caudal clonidine is preferable over caudal opioids (13).

Yildis et al. in 2005 performed a study on 60 children aged 1-10 yrs. who were candidates for elective herniorrhaphy. Patients were divided into 4 groups. In group 1, bupivacaine 0.125%, 1 ml/kg alone and in groups 2, 3 and 4 bupivacaine along with 1, 1.5 and 2 µg/kg clonidine, respectively were used for caudal block. They showed that duration of analgesia was significantly longer in the group receiving 2 µg/kg clonidine and peri-operative hypotension or bradycardia, and post-operative respiratory depression or loss of motion did not occur in any patient (10). These findings were in accord with our study results. In our study, mean duration of post-op analgesia was 162.00 min in group B and 417.50 min in group BC. These rates in Yildis study were 105 and 650 min, respectively with the dose of 2 µg/kg. Wheeler et al. in his study conducted in 2003 on 30 children who were candidates for infra-umbilical surgeries showed that addition of clonidine to bupivacaine in caudal block did not significantly increase the duration of post-op analgesia. In his study he used bupivacaine 0.125% 1 ml/kg and clonidine 2 µg/kg (8). The only difference between Wheeler’s study and those of others was that in his study both groups received epinephrine 2:100,000 as well. Epinephrine reinforces and prolongs the local anesthetic block by inducing vasoconstriction slowing down the elimination of local anesthetic. Clonidine has similar mechanism of action. Considering our study results, we recommend the addition of clonidine (unless in cases where neuraxial techniques are contraindicated) in below the navel pediatric surgeries for longer post-op analgesia in pediatric patients.
